# Brain structural changes and the development of interference control in children with ADHD: The predictive value of physical activity and body mass index

**DOI:** 10.1016/j.nicl.2022.103141

**Published:** 2022-08-04

**Authors:** Sebastian Ludyga, Toru Ishihara

**Affiliations:** a Department of Sport, Exercise and Health, University of Basel, Basel, Switzerland; b Graduate School of Human Development and Environment, Kobe University, Kobe, Japan

**Keywords:** Cortical thickness, Intracortical myelination, Executive function, Physical fitness, Exercise

## Abstract

**Background:**

Children with ADHD face deficits in interference control due to abnormalities in brain structure. A low body mass index and high physical activity are factors promoting brain health and may have the potential to reduce ADHD-related cognitive deficits. We aimed to investigate the predictive values of ADHD, body mass index and physical activity for interference control and the potential mediation of these associations by brain structure.

**Method:**

At 9 and 11 years, 4576 children with ADHD and neurotypical peers from the ABCD-cohort completed a Flanker task, anthropometric assessments and reported physical activity. Additionally, T1- and T2-weighted magnet resonance images were collected at both measurement time points.

**Results:**

ADHD, lower physical activity and higher body mass index at baseline predicted lower interference control. Gray matter volume, surface area and gray-white matter ratio contributed to interference control. The longitudinal association between body mass index and interference control was mediated by gray-white-matter ratio. This mediating effect was stronger for children with ADHD than neurotypical peers and mainly restricted to regions associated with cognitive control.

**Conclusion:**

The maintenance of a lower body mass index contributes to interference control by a tendency to normalize regional alterations in grey-white-matter ratio. Being compliant with physical activity also promises higher interference control, but brain structure does not seem to underlie this association.

## Highlights

• Children with ADHD show deficits in interference control during preadolescence.
• Abnormalities in gray-white matter ratio contributed contribute to these deficits.
• Higher physical activity and lower body mass index predict higher interference control.
• Gray-white matter ratio underlies the predictive value of body mass index.
• Brain structure does not explain the predictive value of physical activity.

## 1 Introduction

A national survey supports a ADHD (Attention Deficit Hyperactivity Disorder) prevalence of 10 % among US children and adolescents (Danielson et al., 2018), with an increasing trend found for the age group of 10–14 years (Sayal et al., 2018). ADHD is characterized by developmentally inappropriate levels of inattention, impulsivity, and/or hyperactivity that manifest before children turn 12 years (Association AP, 2013). These symptoms are linked with deficits in executive function (Brocki et al., 2010, Landis et al., 2021), which predict delayed school readiness (Pellicano et al., 2017), poor academic performance (Samuels et al., 2016), problems with peers (Holmes et al., 2016), and low occupational functioning (Barkley and Fischer, 2011). Impairments in several real-life domains further contribute to long-term differences in earnings and savings (Pelham et al., 2019), highlighting the need for (early) support. Executive function serves as biomarker of ADHD, given that gains in this cognitive domain predict reduced symptom severity over time (Rajendran et al., 2013). In this respect, monitoring of inhibitory control in particular is promising, because it is conceptualized as common executive function, whereas working memory and set-shifting characterize more separable components (Miyake and Friedman, 2012). Interference control is one aspect of inhibitory control that is implicated in ADHD and describes the ability to selectively attend and resist distractions at the level of perception (Mueller et al., 2017). During tasks demanding interference control, children with ADHD show hypo-activation of the inferior frontal cortex (IFC), the anterior cingulate cortex (ACC), the insula and the inferior parietal lobule (IPL) in comparison to neurotypical peers (Hart et al., 2013). This functional abnormality is complemented by abnormalities in brain structure, including altered gray matter volume, surface area, and cortical thickness (Cortese et al., 2012, Hoogman et al., 2019). However, some difficulties to pinpoint the regional convergence of ADHD using these indices in particular have been noted as a consequence of the heterogeneity among patients (Samea et al., 2019). In contrast, the gray-white matter ratio (GWMR), which reflects differential myelination of the cerebral cortex and subjacent white matter, appears to be more sensitive to inter-individual differences in psychopathology among youth (Norbom et al., 2019). This marker further allows the detection of abnormalities in brain maturation, since it accurately predicts biological age and correlates of the cognitive development (Lewis et al., 2018). Moreover, a high intracortical myelination, which can be indexed by a low GWMR, has been found to be a transdiagnostic feature of disinhibition (i.e. a loss of top-down control of behavior) (Romero-Garcia et al., 2021). These findings suggest that GWMR might also be affected in children with ADHD, but previous examinations of brain structure in this clinical population have focused on other indices.

The key symptoms of ADHD and brain abnormalities underline the need for effective treatments. International consensus recommends behavioral approaches as first line treatment in children with ADHD, except for cases with more severe symptoms (Caye et al., 2019, Ropper and Cortese, 2020). Even though physical activity is not yet recognized as an evidence-based medicine in ADHD, it has been suggested as a cost-effective intervention that improves executive function across neurodevelopmental disorders (Ludyga et al., 2021). Interference control in particular seems to be sensitive in ADHD, given that experimental studies consistently support benefits for this cognitive function following structured sports programs (Kadri et al., 2019, Kang et al., 2011, Pan et al., 2016, Verret et al., 2012). The effectiveness of such interventions may in part be due to a general increase in movement time. In comparison to neurotypical peers, children with ADHD are less likely to engage in movement behaviors regularly (Mercurio et al., 2021) and most of them fail to meet the recommended daily amount of 60 min of moderate-to-vigorous physical activity (Tandon et al., 2019). However, compliance with this recommendation should be encouraged, given that there is moderate evidence sopporting cognitive benefits of physical activity in children (Erickson et al., 2019). This can be due to a direct effect of physical activity, but also its role in the management of obesity (Chang et al., 2017, Hsieh et al., 2022). A more sedentary lifestyle in children with ADHD is also reflected in a 40 % higher obesity prevalence than in neurotypical peers (Cortese et al., 2016). This is indicated by an increased body mass index (BMI), which in turn, is related to low executive function and reduced cortical thickness (Ronan et al., 2020, Laurent et al., 2020).

Cognitively enhancing effects of physical activity appear to be crucial during the transition to adolescence, when children with ADHD experience a delay in executive function development (Skogli et al., 2017). A role of physical activity in the promotion of the cognitive maturation is further supported by its effects on brain function and structure. Neuroimaging findings revealed a task-dependent facilitation of activity within regions subserving interference control in neurotypical children (Davis et al., 2011, Krafft et al., 2014). Within this functional unit, gray matter volumes of the prefrontal cortex and anterior cingulate cortex have been found to be sensitive to physical activity in a series of twin studies (Tarkka et al., 2019). Similarly, thinner thickness of the prefrontal cortex also accounts for poor executive function in children with high BMI (Ronan et al., 2020, Laurent et al., 2020). This accords well with a recent review suggesting that obesity causes multiple brain structural dysfunctions, which mainly affect prefrontal- and hippocampal-dependent cognitive functions (Hsieh et al., 2022). Consequently, physical activity and weight status seem to affect brain regions associated with the development of executive function (Dumontheil, 2016) and ADHD-related cognitive impairments (Cortese et al., 2012, Loyer Carbonneau et al., 2021). While this provides a first indication for a moderating role of brain structures, there is a paucity of studies that examine mechanisms by which physical activity and healthy weight benefit executive function in ADHD.

In preadolescent children, we investigated longitudinal associations of ADHD status, physical activity and BMI with interference control. Based on the existing literature, we expected that higher physical activity and lower body mass index predicted better interference control. We further examined whether brain structure (gray matter volume, surface area, cortical thickness, and GWMR) in regions underlying interference control mediated these associations.

## 2 Material and methods

### 2.1 Participants

We used longitudinal data from the multi-centered, ongoing Adolescent Brain & Cognitive Development (ABCD) Study. The ABCD study distributes information material and offers researcher-led presentations at local school to recruit children aged 9–10 years. Our analysis was restricted to ADHD children (based on *Diagnostic and Statistical Manual of Mental Disorders*, *Fifth Edition* [DSM-5] criteria) (Association AP, 2013) and neurotypical peers (*N* = 4576) that completed both the baseline and 2-year follow-up assessments (Table 1). The institutional review boards of the University of California, San Diego (IRB# 160091) and the local study sites (*N* = 22) approved the study protocol. Children provided verbal assent and written informed consent was obtained from their parents and/or caregivers. The reporting of the study is in line with the Strengthening the Reporting of Observational Studies in Epidemiology (STROBE) checklist (Table A.1).

**Table 1:** Participants’ characteristics, physical health and cognitive performance at baseline and follow-up.

	ADHD (*N* = 173 f / 429 m)				Neurotypical (*N* = 2004 f / 1970 m)			
	Baseline		Follow-up		Baseline		Follow-up	
Right handedness	448*	(74 %)			3204	(81 %)		
Age in m	119.1	(7.5)			119.6	(7.4)		
Scan time interval in m	23.7	(1.6)			23.8	(1.6)		
Family income^a^	7.3*	(2.3)			7.5	(2.2)		
Parent’s education^b^	16.9	(2.3)			16.8	(2.7)		
Partner’s education^b^	16.5	(2.6)			16.5	(2.9)		
Puberty rating^c^	2.1	(.8)			2.1	(.8)		
Sleep total score^d^	36.5	(5.8)			36.5	(5.8)		
Vision abilities^e^	6.8	(1.5)			6.9	(1.5)		

Height in cm	139.8*	(8.3)	151.9*	(8.9)	141.0	(7.9)	153.4	(8.8)
Weight in kg	36.1*	(9.7)	47.2*	(13.4)	37.8	(10.2)	49.3	(14.0)
BMI in kg^.^m^−2^	18.3*	(3.8)	20.3*	(4.6)	18.9	(4.0)	20.8	(4.8)
Physical activity ≥ 60 min (days/week)	3.2*	(2.5)	3.6*	(2.2)	3.6	(2.3)	3.9	(2.1)
Score on Flanker task	92.8*	(10.0)	99.0*	(8.1)	94.9	(8.5)	100.5	(7.3)

*Notes:* * *p* < .05 versus neurotypical children (χ^2^ or unpaired t-tests). ^a^1 = Less than $5,000; 2 = $5,000 through $11,999; 3 = $12,000 through $15,999; 4 = $16,000 through $24,999; 5 = $25,000 through $34,999; 6 = $35,000 through $49,999; 7 = $50,000 through $74,999; 8 = $75,000 through $99,999; 9 = $100,000 through $199,999; 10 = $200,000 and greater. ^b^0 = Never attended/Kindergarten only; 1 = 1st grade; 2 = 2nd grade; 3 = 3rd grade; 4 = 4th grade; 5 = 5th grade; 6 = 6th grade; 7 = 7th grade 8 = 8th grade; 9 = 9th grade; 10 = 10th grade; 11 = 11th grade; 12 = 12th grade; 13 = High school graduate; 14 = GED or equivalent Diploma General; 15 = Some college; 16 = Associate degree: Occupational; 17 = Associate degree: Academic Program; 18 = Bachelor’s degree; 19 = Master’s degree; 20 = Professional School degree; 21 = Doctoral degree. ^c^Assessed by ABCD Youth Pubertal Development Scale and Menstrual Cycle Survey History (low-prepuberty, high-puberty). ^d^Assessed by ABCD Parent Sleep Disturbance Scale for Children low-good sleep, high-poor sleep. ^e^Assessed by ABCD Youth Snellen Vision Screener (low-poor vision, high-good vision).

### 2.2 Procedures

The ABCD study examines children aged 9 to 10 years and follows them up 10 years into young adulthood. Longitudinal data is collected on physical health, mental health, neurocognition, brain function and structure, substance abuse, culture and environment as well as other aspects. For the present analysis, we selected data from the baseline assessment and two-year follow-up Procedures during both assessment time points were standardized and identical across all study sites. This was further supported by the requirement that each site had to have the research expertise and the equipment to collect data according to the ABCD study protocol (Casey et al., 2018, Barch et al., 2018). Preselected variables of interest were ADHD diagnosis, BMI, physical activity, interference control and indices of brain structure. Family income, parents’ education, puberty ratings, sleep and vision at baseline served as potential confounders.

### 2.3 ADHD diagnosis

Parents or caregivers of study participants completed a computerized version of the Kiddie Schedule for Affective Disorders and Schizophrenia for DSM-5 (Barch et al., 2018). For current episode diagnosis, a high concordance between the computerized and traditional versions has been found (88–96 % agreement) (Townsend et al., 2020). Children, who fulfilled the DSM-5 criteria for ADHD at baseline (not including cases with partial remission), were considered cases and those with no DSM-5 diagnosis served as neurotypical controls.

### 2.4 Cognitive assessment

In the Flanker Inhibitory Control and Attention Test of the National Instruments of Health Cognition Toolbox, a central target arrow flanked by two similar stimuli on each side (left and right) was presented on an iPad (Apple, USA, California, Cupertino). Depending on the trial type, the flanking arrows faced in the same (congruent) or different direction (incongruent) compared to the target arrow. Participants were instructed to indicate the direction of the central stimulus by pressing an on-screen button corresponding to left or right. Following a fixation period (random variation between 1000 and 1500 ms), a cue (1000 ms) reminded participants to focus on the central arrow. The presentation of the flanking arrows commenced 100 ms before the central arrow and the whole test stimulus remained onscreen over 10000 ms or a response was given. When participants responded correctly on 75 % of the practice trials, they advanced to the test items. Otherwise, two additional practice blocks were administered. During practice, a voice prompted participants to correct their response, if an incorrect response was given. The subsequent test block consisted of 16 congruent and 9 incongruent trials, which were presented in pseudorandom order (with 1 to 3 congruent trials preceding each incongruent trial). Scoring of the Flanker task was based on accuracy (when less than 80 % trials were responded correctly) or accuracy and reaction time (when at least 80 % trials were responded correctly). The two-vector method and equations underlying the calculation of the score has been described in a previous validation study (Zelazo et al., 2013).

### 2.5 Physical health

Participants completed the Youth Risk Behavior Survey (Center for Disease Control, 2016) and for the present study, we only included the total number of days/week with physical activity ≥ 60 min/day. Both weight and height were assessed three times during a single laboratory visit. The two closest or all three measurements (only when the third measurement fell equally between the two other ones) were averaged.

### 2.6 MRI data acquisition and processing

Fully preprocessed morphometric and image intensity measures were provided by the ABCD study (Hagler et al., 2019). T1- and T2-weighted images were acquired using 3-T MRI scanners. The imaging data obtained from three manufacturers’ scanner platforms were harmonized. During imaging acquisition, a child friendly movie was played on the screen.

**Fig. 1:**
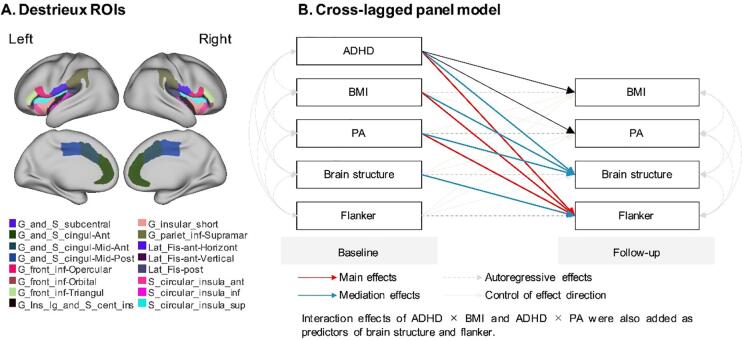
Loci of the brain regions (A) and cross-lagged panel model used in this study (B). ROI = Region of interest. Notes: ADHD = Attention Deficit Hyperactivity Disorder; BMI = Body mass index; PA = Physical activity.

The MRI data were processed using FreeSurfer v5.3 to obtain morphometric (cortical thickness, area, volume, and sulcal depth) and image intensity measures (T1- and T2-weighted gray-white ratio) for each of the 148 Destrieux atlas regions of interest (ROIs). For the present analysis, we focused on 16 ROIs in each hemisphere: ACC, midcingulate cortex (MCC), IFC, insula, and IPL (Fig. 1). Our selection was based on *meta*-analytical findings highlighting regions sensitive to ADHD-related deficits in interference control (Hart et al., 2013). For the calculation of gray-white matter ratio ([white - gray] / [white + gray] / 2), we used intensity values at a distance of .2 mm relative to the gray-white boundary.

### 2.7 Statistical analyses

All statistical analyses were conducted with R Studio (version 1.1.463). For the examination of longitudinal associations within the cross-lagged panel (Fig. 1), we used the sem function in the lavaan package. Our first model investigated the association between baseline ADHD status (coded 0 = neurotypical; 1 = ADHD), physical activity, BMI and follow-up Flanker task performance, while controlling for autoregressive effects (baseline scores). As we planned to use continuous data rather than categories for BMI, a preliminary analysis used curve fitting to test whether its association with interference control followed a linear or non-linear trend and the term was adjusted, if necessary. When the initial model indicated longitudinal associations between one or more predictors and the outcome, their mediation by MRI indices were examined in a second model. Only MRI indices that significantly (false discovery rate [FDR] corrected p < .10) predicted Flanker task performance were included. In addition to the prediction of MRI indices from ADHD status, physical activity and BMI, their interaction terms (ADHD × BMI; ADHD × physical activity; BMI × physical activity) were also included into the model to examine moderated mediations. Interaction effects were followed-up by post-hoc analyses testing the prediction of MRI indices from BMI and physical activity separately in children with ADHD and neurotypical peers. For indices with a mediating effect, reverse causations were tested by exchanging predictors and outcomes. The level of significance was set to p < .05. The lm and sim_slopes functions were used to adjust regressions for scan time interval, scanner platforms, age, sex, handedness, parents’ educational history, family income, pubertal status, sleep status and vision. Missing variables were handled by full-information maximum likelihood estimation. Absolute and incremental fit indices were calculated for each model and considered good at RMSEA < .06 and CFI > .95 (Xia and Yang, 2019).

## 3 Results

### 3.1 Behavioral performance

ADHD (β = −.04, p = .007), lower physical activity (β = .06, p < .001) and higher BMI (β = −.07, p < .001) at baseline predicted lower interference control at follow-up. The association between BMI (see Table B.1 for the distribution of BMI percentiles) and interference control was linear and not better explained by a quadratic (β = −.007, p = .29) or other non-linear trend. The interactions of ADHD status with physical activity (β = −.02, p = .13) and with BMI (β = −.01, p = .43) as well as the interaction of physical activity and BMI (β = −.003, p = .84) did not reach a statistically significant level and remained unchanged after controlling for confounders (Table B.2). The initial adjusted and unadjusted models showed good model fit (RMSEA ≤ .05; CFI ≥ .96).

### 3.2 Brain structure

**Fig. 2:**
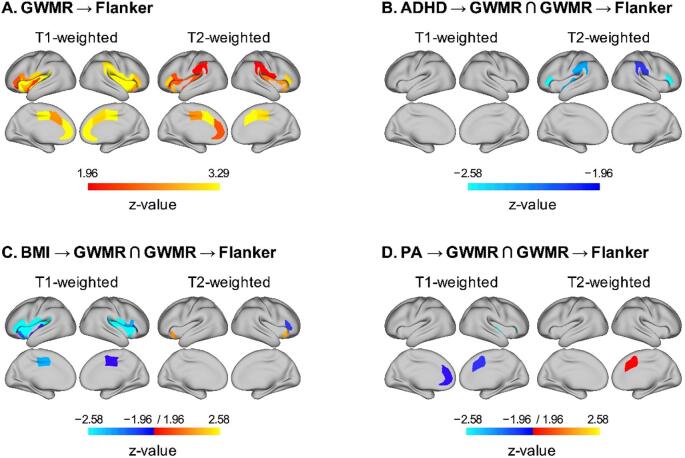
Prediction of Flanker task performance by T1- and T2-weighted gray-white-matter ratio (FDR corrected p < .10) (A) and mediation effects of gray-white matter ratio on the prediction Flanker task performance by baseline ADHD (B), BMI (C), and PA (D). Notes: ADHD = Attention Deficit Hyperactivity Disorder; GWMR = Gray-white matter ratio; BMI = Body mass index; PA = Physical activity.

When MRI data and confounders were added, interference control at follow-up was associated with T1 GWMR (31 regions), T2 GWMR (22 regions), surface area (14 regions) and grey matter volume (3 regions) (Fig. 2A), but not with sulcul depth and cortical thickness. All models showed good model fit (RMSEA ≤ .03; CFI ≥ .96).

**Fig. B.1:**
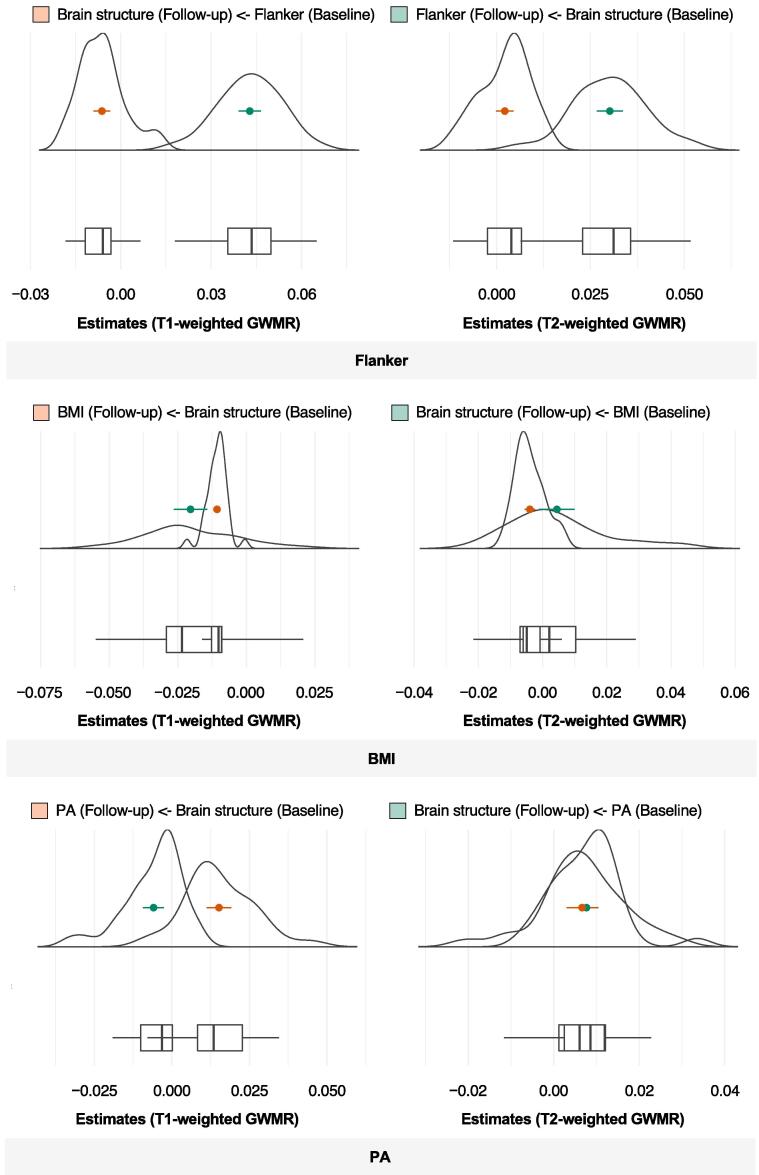
Models testing the reverse causation, predicting follow-up body mass index (BMI) and physical activity (PA) from baseline gray-white-matter ratio (GWMR), and predicting follow-up GWMR from baseline Flanker performance. Notes: Each plot indicates standardized coefficients for each region.

Low T2 GWMR in bilateral IFC, IPL, and left insula partly mediated the relation between ADHD and interference control at follow-up (Fig. 2B). Moreover, T1 GWMR in bilateral MCC, IFC, and insula as well as T2 GWMR and surface area in right IFC (Fig. 2C) mediated the association of BMI and interference control at follow-up. However, the opposite direction of coefficients were found for T2 GWMR in bilateral anterior insula. The association of physical activity and interference control at follow-up was mediated by T2 GWMR in left ACC and surface area in right IFC and insula (Fig. 2D). In contrast, the direction of coefficients was reversed for T1 GWMR in bilateral ACC and right insula. Models testing reverse causations did not support that baseline GWMR predicted physical activity and BMI at follow-up (Fig. B.1).

**Fig. 3:**
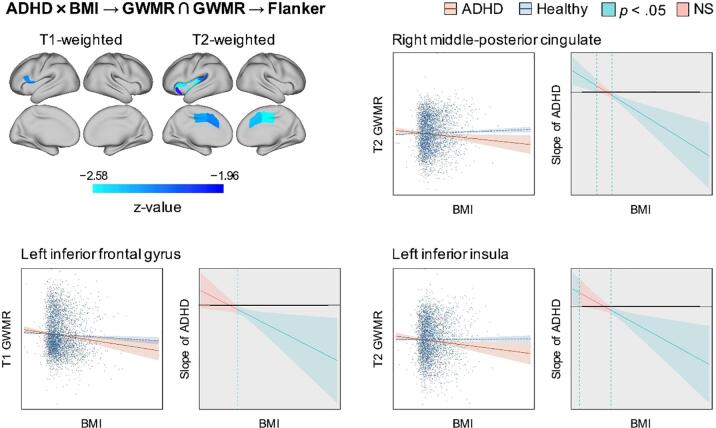
Moderating effect of ADHD on the mediation of longitudinal associations of body mass index and Flanker task performance by gray-white-matter ratio. Notes: To indicate the direction of effects, only three regions are shown as representative examples. ADHD = Attention Deficit Hyperactivity Disorder; GWMR = Gray-white matter ratio; BMI = Body mass index.

**Fig. B.2:**
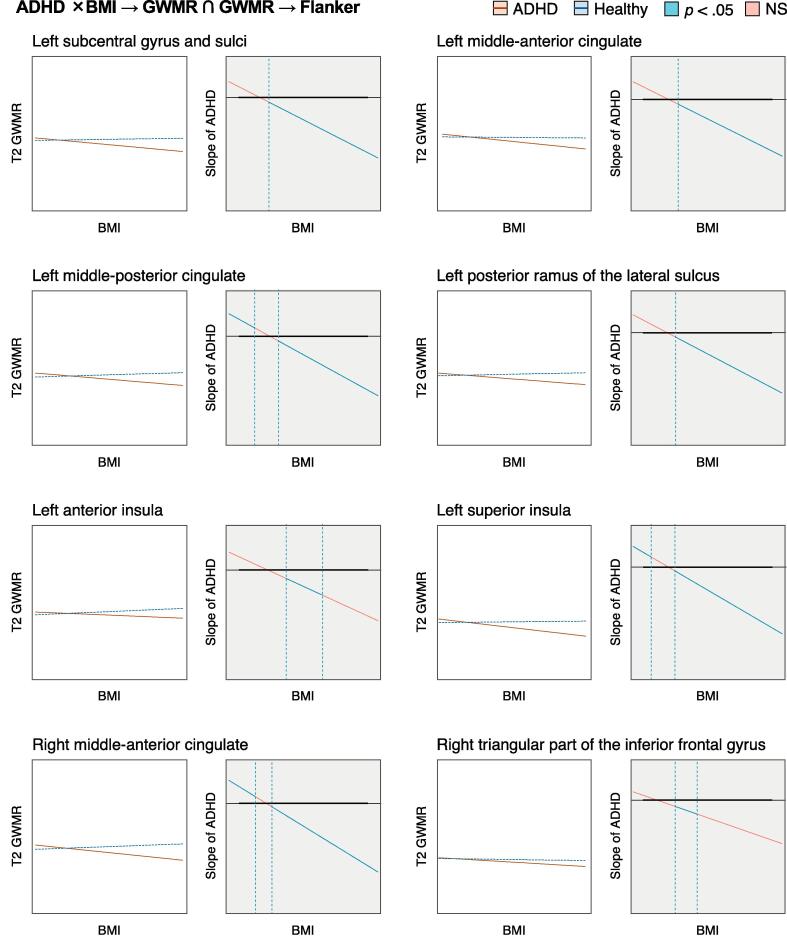
Moderating effect of ADHD on the mediation of longitudinal associations of body mass index and Flanker task performance by gray-white-matter ratio across multiple regions. Notes: ADHD = Attention Deficit Hyperactivity Disorder; GWMR = Gray-white matter ratio; BMI = Body mass index.

**Fig. B.3:**
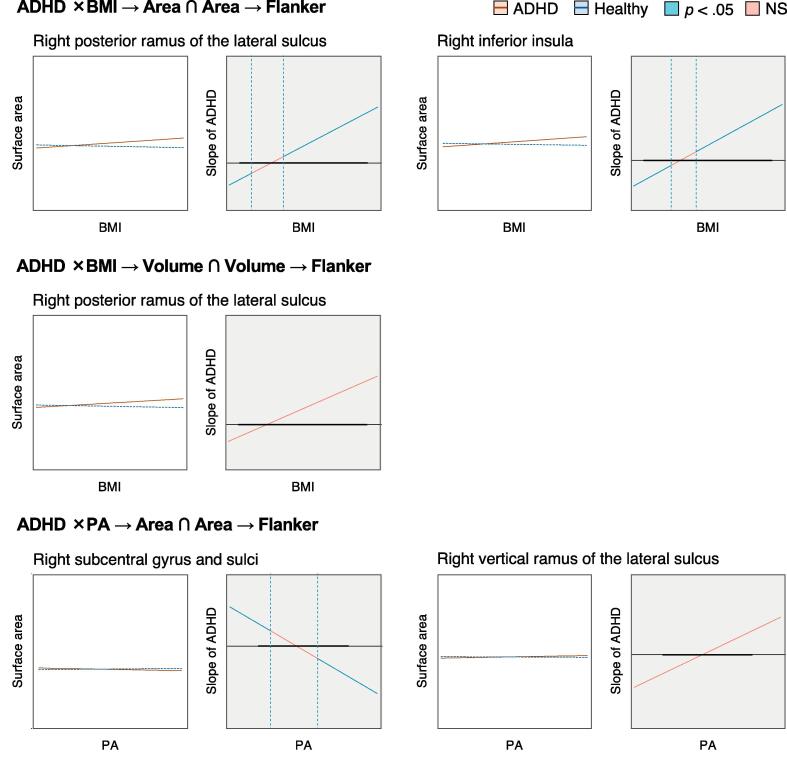
Moderating effect of ADHD on the mediation of longitudinal associations of body mass index, physical activity and Flanker task performance by surface area and gray matter volume across multiple regions. *Notes:* ADHD = Attention Deficit Hyperactivity Disorder; BMI = Body mass index; PA = Physical activity.

Moderated mediation analyses revealed interactions of ADHD status with BMI for T1 GWMR (1 region) and T2 GWMR (10 regions) (Fig. B.2). Post-hoc analyses supported T1 GWMR in left IFC and T2 GWMR in bilateral ACC and MCC as well as (left anterior, inferior, and superior) insula to mediate the association of higher BMI with lower interference control at follow-up in children with ADHD only (Fig. 3). Moderated mediation analyses also revealed significant interactions of ADHD status with physical activity for surface area and gray matter volume, but the direction of coefficients was inconsistent (Fig. B.3).

## 4 Discussion

Compared to neurotypical peers, children with ADHD performed worse on the Flanker task at follow-up, partly due to regional GWMR abnormalities. In both groups, lower physical activity and higher BMI at baseline independently predicted lower interference control after two years, when autoregressive effects were accounted for. Regression coefficients further indicated that both variables individually explained a proportion of variance in interference control that was similar or even greater than the proportion that was attributed to the ADHD diagnosis. Alterations in GWMR partly accounted for the predictive value of BMI in children with ADHD, but did not underlie the association of physical activity and interference control.

In ADHD, gains in interference control are observable in early years (Suades-González et al., 2017), but the developmental progress is slowed down in subsequent years and causes executive function deficits to appear more prominent during preadolescence (Skogli et al., 2017, Tillman et al., 2015). Our results support that children with ADHD still showed lower interference control than neurotypical peers at follow-up. Even though neuroimaging evidence suggests that children with ADHD face abnormalities in grey matter volume, cortical thickness and surface area across several regions, these indices did not account for an impaired interference control. In contrast, GWMR in bilateral IFC, IPL and left insula partly explained these ADHD-related deficit.

The low GWMR we found in children with ADHD reflects more similar gray and white matter signal intensity. A similar pattern was found in pre- and postcentral cortices as well as parts of the frontal cortex in children showing mental problems and low cognitive ability (Norbom et al., 2019). Individual differences in psychopathology may be sensitive to GWMR as it is inversely associated with intracortical myelin and myelin-based water content on T1 and T2 images, respectively. Higher association cortices are less myelinated compared to primary association cortices (Glasser et al., 2013). Studies tracking GWMR across age showed that there is an ongoing intracortical myelination that extends past adolescence (Grydeland et al., 2013), with a protracted decrease of GWMR in association cortices in particular characterizing normal structural brain maturation (Lewis et al., 2018, Westlye et al., 2010). The low GWMR in IFC and IPL indicates an atypically high intracortical myelination in children with ADHD. The regional specificity and direction of coefficients render a catch-up effect likely, but interference control still differed between groups at follow-up. This is further supported by T1/T2 ratio findings indicating an increase of intracortical myelination with age, but an inverse association with general cognitive ability in regions including frontal and parietal cortices (Norbom et al., 2020). An atypically high level of intracortical myelin may cause detrimental effects on cognitive performance due to its ability to inhibit synapse formation and to decrease neuronal plasticity (Snaidero and Simons, 2017). Additionally, deficits in interference control could also be linked with altered network activity, given that functional connectivity is higher between areas with similar intracortical myelin levels (Huntenburg et al., 2017). Children with ADHD showed low GWMR in IFC and IPL, but low GWMR is only expected for primary association cortices in this age group (Glasser et al., 2013) The link between network activity and similar intracortical myelination might partly explain profiles of ADHD-related over-connectivity during the Flanker task (Michelini et al., 2019).

Abnormalities in GWMR further accounted for the prediction of interference control by BMI. Despite the linear relation between these variables, the low proportion of participants in the 5th percentile range and a high proportion of participants in the 95th percentile range provides an indication that overweight and obesity have detrimental effects on GWMR. The mediating role of GWMR was more pronounced in children with ADHD compared to neurotypical peers. Previous findings have shown that young adults with a higher BMI show a variety of brain abnormalities, including increased intracortical myelination in regions involved in somatosensory processing and inhibitory control (Dong et al., 2021). We extend these findings by showing that low GWMR in left IFC, bilateral ACC, MCC and insula partly accounted for impaired interference control in children with ADHD. This may be due to the consequences of structural abnormalities for underlying brain functions. IFC, ACC and MCC form parts of the cognitive control network and are recruited when faced with inhibitory demands (Niendam et al., 2012). Evidence from source imaging suggests that specifically the ACC contributes to behavioral performance on the Flanker task by its role in the monitoring and the detection of conflict induced by incongruent stimuli (Siemann et al., 2016). However, the mediating effect of GWMR may extent to other cognitive functions, given that the ACC has been proposed to optimize the allocation of cognitive control based on an assessment of the overall expected value of control (Shenhav et al., 2016). The insula is characterized by a task-independent hyperactivation that often expands to the ACC and likely reflects autonomous nervous system response to cognitive challenge (Gasquoine, 2014). Due to the modulation of neuronal activity by intracortical myelination (de Faria et al., 2021), a higher BMI might influence interference control by compromising the function of its underling neural networks. As ADHD has been considered as both a cause and consequence of weight gain (Cortese and Tessari, 2017), the association of GWMR abnormalities and cognitive function underlines the need to monitor the patients’ BMI. This is further supported by the observation that some pharmacological treatments applied in ADHD elicit further increases of the BMI (Gurka et al., 2021). While physical activity has the potential to influence executive function by normalizing the BMI (Chang et al., 2017), it was independently associated with interference control in children with ADHD and neurotypical peers. Both GWMR and surface area influenced this association, but the direction of coefficients were inconsistent. Consequently, physical activity seems to promote interference control by mechanisms that have not been examined in our study.

Even though our findings provide first indications on pathways by which BMI influences interference control in children with ADHD, their interpretation is limited by a very low strength of the interrelations. Major factors contributing to less pronounced differences in behavioral performance between patients with ADHD and neurotypical peers include a restriction of the study period to only two years and specific recruitment procedures. In the ABCD study, children with externalizing and/or internalizing problems were over-represented at baseline (Garavan et al., 2018), but not all of them necessarily were diagnosed with a neurodevelopmental disorder. This increases the chance that the neurotypical group included children with no DSM-5 diagnosis, but executive function deficits (Oh et al., 2020). Despite the low strength of interrelations, they can still be meaningful. In this respect, the use of conventional effect sizes for drawing conclusions in psychological research has been criticized (Schäfer and Schwarz, 2019), giving rise to an alternative approach that focuses on whether effect sizes were estimated reliably. We employed a cross-lagged panel that investigated (moderated) mediation effects in a large cohort, suggesting that even very small effects can be considered consequential (Funder and Ozer, 2019). Our results indicate that BMI and physical activity predict interference control in children with ADHD across two years. Consequently, both variables have an influence on the severity of deficits, but direct conclusions on whether changes in BMI and physical activity (induced by interventions) may alter their prognosis cannot be drawn directly from our results. However, this aspect can be addressed by using longitudinal modelling of changes on the ABCD cohort, when full data from at least three measurement time points becomes available. Another limitation of the present analysis is the focus on the number of days compliant with the physical activity recommendations, which represents only a quantitative measure of physical activity. Thus, the predictive value of the type of movement behaviors for performance on the Flanker task remains unclear. Moreover, the current study was limited to interference control, although deficits in other components of executive function are also evident in ADHD (Kofler et al., 2019). For GWMR, mediating effects were found in regions that are also activated during cognitive tasks tapping into set-shifting and working memory (Duma et al., 2019, Bissonette et al., 2013). Thus, the BMI may have the potential to elicit more general benefits for executive function by altering GWMR.

## 5 Conclusions

During the transition from childhood to adolescence, children with ADHD show lower interference control relative to neurotypical peers due to abnormalities in brain structure. Compared to the ADHD diagnosis, a higher BMI and lower physical activity seem to have at least the same predictive value for this cognitive function. The prevention of a higher BMI has a positive effect on inteference control as it tends to normalize ADHD-related alterations in brain structure. Consequently, practitioners may encourage the monitoring of weight status and physical activity levels to predict deficits in interference control and eventually support the ADHD treatment in children.

## CRediT authorship contribution statement

**Sebastian Ludyga:** Conceptualization, Data curation, Investigation, Methodology, Project administration, Resources, Validation, Writing – original draft. **Toru Ishihara:** Conceptualization, Data curation, Formal analysis, Investigation, Methodology, Project administration, Resources, Validation, Visualization, Writing – review & editing.

## Declaration of Competing Interest

The authors declare that they have no known competing financial interests or personal relationships that could have appeared to influence the work reported in this paper.

## Figures and Tables

**Table A.1:** STROBE checklist.

	Item No	Recommendation	Page No
Title and abstract	1	(*a*) Indicate the study’s design with a commonly used term in the title or the abstract	1
		(*b*) Provide in the abstract an informative and balanced summary of what was done and what was found	2

**Introduction**			
Background/rationale	2	Explain the scientific background and rationale for the investigation being reported	4–6
Objectives	3	State specific objectives, including any prespecified hypotheses	6

**Methods**			
Study design	4	Present key elements of study design early in the paper	6–7
Setting	5	Describe the setting, locations, and relevant dates, including periods of recruitment, exposure, follow-up, and data collection	6–7
Participants	6	(*a*) *Cohort study*—Give the eligibility criteria, and the sources and methods of selection of participants. Describe methods of follow-up *Case-control study*—Give the eligibility criteria, and the sources and methods of case ascertainment and control selection. Give the rationale for the choice of cases and controls *Cross-sectional study*—Give the eligibility criteria, and the sources and methods of selection of participants	6–7
		(*b*) *Cohort study*—For matched studies, give matching criteria and number of exposed and unexposed *Case-control study*—For matched studies, give matching criteria and the number of controls per case	NA
Variables	7	Clearly define all outcomes, exposures, predictors, potential confounders, and effect modifiers. Give diagnostic criteria, if applicable	7–8
Data sources/measurement	8*	For each variable of interest, give sources of data and details of methods of assessment (measurement). Describe comparability of assessment methods if there is more than one group	7–8
Bias	9	Describe any efforts to address potential sources of bias	7–8
Study size	10	Explain how the study size was arrived at	8–9
Quantitative variables	11	Explain how quantitative variables were handled in the analyses. If applicable, describe which groupings were chosen and why	8–9
Statistical methods	12	(*a*) Describe all statistical methods, including those used to control for confounding	9
		(*b*) Describe any methods used to examine subgroups and interactions	9
		(*c*) Explain how missing data were addressed	9
		(*d*) *Cohort study*—If applicable, explain how loss to follow-up was addressed *Case-control study*—If applicable, explain how matching of cases and controls was addressed *Cross-sectional study*—If applicable, describe analytical methods taking account of sampling strategy	9
		(*e*) Describe any sensitivity analyses	NA

**Results**			
Participants	13*	(a) Report numbers of individuals at each stage of study—eg numbers potentially eligible, examined for eligibility, confirmed eligible, included in the study, completing follow-up, and analysed	8–9
		(b) Give reasons for non-participation at each stage	NA
		(c) Consider use of a flow diagram	NA
Descriptive data	14*	(a) Give characteristics of study participants (eg demographic, clinical, social) and information on exposures and potential confounders	Table 1
		(b) Indicate number of participants with missing data for each variable of interest	Table 1
		(c) *Cohort study*—Summarise follow-up time (eg, average and total amount)	Table 1
Outcome data	15*	*Cohort study*—Report numbers of outcome events or summary measures over time	Table 1; Fig. 1, Fig. 2, Fig. 3
		*Case-control study—*Report numbers in each exposure category, or summary measures of exposure	*–*
		*Cross-sectional study—*Report numbers of outcome events or summary measures	*–*
Main results	16	(*a*) Give unadjusted estimates and, if applicable, confounder-adjusted estimates and their precision (eg, 95 % confidence interval). Make clear which confounders were adjusted for and why they were included	Appendix
		(*b*) Report category boundaries when continuous variables were categorized	NA
		(*c*) If relevant, consider translating estimates of relative risk into absolute risk for a meaningful time period	NA
Other analyses	17	Report other analyses done—eg analyses of subgroups and interactions, and sensitivity analyses	Appendix

**Discussion**			
Key results	18	Summarise key results with reference to study objectives	12
Limitations	19	Discuss limitations of the study, taking into account sources of potential bias or imprecision. Discuss both direction and magnitude of any potential bias	14–15
Interpretation	20	Give a cautious overall interpretation of results considering objectives, limitations, multiplicity of analyses, results from similar studies, and other relevant evidence	12–14
Generalisability	21	Discuss the generalisability (external validity) of the study results	15

**Other information**			
Funding	22	Give the source of funding and the role of the funders for the present study and, if applicable, for the original study on which the present article is based	15

**Table B.1:** Distribution of the body mass index (BMI) percentiles (adjusted for age and sex) in participating boys and girls at baseline.

	N boys	N girls
Underweight (<5th percentile)	85	48
Healthy weight (5th percentile)	1332	1654
Overweight (85th percentile)	345	352
Obese (95th percentile)	383	325

**Table B.2:** The results of cross-lagged panel model investigating the longitudinal association between ADHD, physical activity, body mass index, and Flanker task performance.

Dependent variables	Independent variables	Unadjusted model				Adjusted model			
		B	Z	Lower CI	Upper CI	B	Z	Lower CI	Upper CI
Physical activity	Flanker	0.03	1.93	0.00	0.06	0.01	0.39	−0.02	0.04
	ADHD	−0.04	−2.52	−0.06	−0.01	−0.04	−3.02	−0.07	−0.02
	Physical activity	0.25	17.45	0.22	0.28	0.23	15.83	0.20	0.26
	BMI	−0.06	−3.94	−0.09	−0.03	−0.03	−1.71	−0.06	0.00

BMI	Flanker	−0.02	−2.34	−0.03	0.00	−0.01	−1.16	−0.02	0.01
	ADHD	0.00	0.19	−0.01	0.02	0.01	0.71	−0.01	0.02
	Physical activity	−0.02	−2.17	−0.03	0.00	−0.01	−1.10	−0.02	0.01
	BMI	0.87	117.14	0.85	0.88	0.85	110.20	0.84	0.87

Flanker	Flanker	0.41	30.55	0.38	0.44	0.39	28.08	0.36	0.41
	ADHD	−0.04	−2.71	−0.06	−0.01	−0.04	−2.79	−0.07	−0.01
	Physical activity	0.06	4.41	0.03	0.09	0.04	2.83	0.01	0.07
	BMI	−0.07	−5.21	−0.10	−0.04	−0.04	−2.50	−0.06	−0.01
	ADHD × Physical activity	−0.02	−1.52	−0.04	0.01	−0.02	−1.35	−0.04	0.01
	ADHD × BMI	−0.01	−0.79	−0.04	0.02	−0.01	−0.87	−0.04	0.02

*Notes:* ADHD = Attention Deficit Hyperactivity Disorder; BMI = Body mass index; PA = Physical activity; CI = confidence interval.

## Data Availability

The present article used data from the ABCD cohort, which can be accessed using the NIMH data archive (https://nda.nih.gov/abcd).

## Appendix A

See

## Appendix B

See

See
